# Difference between Creosote and Carbolic Acid

**Published:** 1887-08

**Authors:** 


### Difference Between Creosote and Carbolic Acid.

Creosote is a distillation from wood tar, carbolic acid from tar of mineral coal; creosote is an oil, carbolic acid an alcohol; creosote is a non-crystallizable fluid, carbolic acid in its pure state is always crystallized except when quite warm; creosote is not soluble in water, carbolic acid is; creosote is not a caustic, carbolic acid is a powerful caustic; creosote is not a germicide, carbolic acid is.—*Mat. Med. and Phar.*

### Resolutions Adopted by Chicago Dental Club, June 27, 1887.

WHEREAS, At the last annual meeting of the American Medical Association held in this city, a resolution was passed recognizing the graduates of all Dental Colleges which require a thorough course of instruction in all the fundamental sciences which underlie medicine and surgery, equal to the standard required by the best medical colleges of our land, as entitled to membership in this Association with all its privileges; and,

WHEREAS, Our honored friends, Drs. N. S. Davis and W. W. Allport, of Chicago, were largely instrumental in bringing about this action of the Association; and,

WHEREAS, Both of these gentlemen have always taken an active interest in promoting higher education among dentists, and were also largely instrumental in the abolishment of dental chairs in the medical colleges of our city, and the section of Dental and Oral Surgery in the International Medical Congress; therefore, be it—

*Resolved*, That we, the members of the Chicago Dental Club, express our appreciation of their untiring efforts and devotion to the cause of higher professional education and the elevation of our "specialty" to the position which its scientific attainments deserve. [Signed]

JOHN S. MARSHALL,<br>A. E. BALDWIN,<br>J. AUSTIN DUNN, } *Committee.*

